# Moral distress and turnover intention in deceased donor organ procurement teams in Serbia: a cross-sectional study

**DOI:** 10.1186/s12910-026-01533-0

**Published:** 2026-06-29

**Authors:** Dragan Nikolić, Janko Pasternak, Vladimir Manojlović, Slavko Budinski, Marijana Basta Nikolić, Nikola Batinić

**Affiliations:** 1 Faculty of Medicine, University of Novi Sad, Novi Sad, Serbia; 2 Clinic for Vascular and Endovascular Surgery, University Clinical Centre of Vojvodina, Hajduk Veljkova 1–3, Novi Sad, 21000 Serbia; 3 Centre for Radiology, University Clinical Centre of Vojvodina, Novi Sad, Serbia

**Keywords:** Moral distress, Deceased donor, Organ procurement, Transplantation, MDS-R, Vascular surgery, Turnover intention, Serbia

## Abstract

**Background:**

Deceased donor organ procurement presents distinct ethical challenges for transplant teams, yet moral distress among these professionals remains understudied, particularly in low-donation settings. This study measured moral distress levels among organ procurement team members at a Serbian tertiary centre and examined factors associated with distress intensity.

**Methods:**

This cross-sectional study administered a transplantation-adapted Moral Distress Scale-Revised (MDS-R) to 22 active transplant team members at the University Clinical Centre of Vojvodina in 2023 (response rate 78.6%, 22/28). The adapted Serbian-language instrument demonstrated excellent internal consistency (Cronbach’s α = 0.89); however, it has not undergone full psychometric validation for transplantation contexts. We analysed MDS-R total composite scores (frequency × intensity, range 0–336) across professional groups and examined correlations with demographic and professional variables using non-parametric statistics.

**Results:**

Mean MDS-R total composite score was 82.4 ± 31.8 (range 38–132), suggesting substantial moral distress in this sample. Compared with published point estimates from ICU (69.5) and emergency settings (71.2), our estimate was numerically higher, but the 95% CI overlapped both reference values; cross-study comparisons are descriptive only. Significant differences emerged across professional groups (Kruskal–Wallis H = 8.73, df = 4, *p* = 0.013, η² = 0.28): vascular surgeons reported the highest observed scores (96.4 ± 27.3), though with only four participants this finding requires cautious interpretation. Post-hoc analysis revealed a significant difference between vascular surgeons and transplant coordinators (adjusted *p* = 0.018). Years of transplantation experience correlated negatively with distress (Spearman’s ρ = − 0.47, 95% bootstrap CI [–0.73, − 0.12], *p* = 0.02). Highest-intensity distress items in the full sample were family communication dilemmas (3.5 ± 0.8) and donor body objectification concerns (3.4 ± 0.9). Nearly 60% of participants reported having considered leaving transplantation; intention to leave correlated with MDS-R scores (Spearman’s ρ = 0.52, bootstrap 95% CI [0.18, 0.76], *p* = 0.01), although reasons for considering departure were not systematically collected.

**Conclusions:**

Organ procurement team members in this low-donation setting reported substantial moral distress. While vascular surgeons had the highest observed scores, this finding may reflect role-specific factors, demographic differences, or other unmeasured confounders, and requires confirmation in larger samples. The association between moral distress and self-reported intention to leave transplantation, though exploratory, highlights the need for further investigation of retention-focused support strategies in procurement teams.

**Supplementary Information:**

The online version contains supplementary material available at https://doi.org/10.1186/s12910-026-01533-0.

## Background

Organ transplantation has become an established component of contemporary clinical practice, but the preceding step—retrieving organs from a donor declared dead by neurological criteria while physiological support is temporarily maintained—remains ethically complex and emotionally demanding for clinicians in the procurement theatre. The concept of moral distress—psychological disequilibrium arising when one knows the ethically appropriate action but feels constrained from taking it—was first defined by Jameton in 1984 and has since been extensively studied in intensive care and oncology settings [1, 2]. Moral distress has been widely reported among clinicians in intensive care and emergency settings, with prevalence varying across studies and instruments [3, 4]. Deceased donor organ procurement presents specific challenges that may generate distinct patterns of distress, particularly for team members who directly perform explantation procedures.

In Serbia, our centre performs on average four deceased donor kidney transplants per year, corresponding to a national donation rate of about 5.2 donors per million population (pmp)—far below the EU average of 20.9 and Spain’s 49.4 pmp [5, 6]. Serbia’s low deceased-donor rate suggests that barriers to donation are not solely technical or clinical. In a low-donation setting, procurement cases are relatively infrequent and may be experienced as particularly consequential by the teams involved.

The ethical complexity of deceased donor organ procurement is particularly pronounced for surgeons who directly perform explantation procedures. Although brain death is well understood by transplant professionals on the basis of clear clinical criteria established by the World Brain Death Project [7], the concept remains challenging for families and the broader public [8, 9]. For procurement team members, the practical reality of performing organ retrieval from a donor with maintained circulation and apparent vital signs—despite confirmed brain death—may impose a distinct psychological burden. Empirical studies have documented emotional distress and negative attitudes among operating room staff involved in organ procurement [10]. Such distress may arise both from the nature of the explantation procedure itself and from the interpersonal context surrounding donation—including real or anticipated conflict with grieving families—and limited exposure to these infrequent cases may heighten its impact; these sources are not mutually exclusive.

Most published work on moral distress has focused on ICU and nursing settings, whereas procurement teams have received far less attention. Epstein and Hamric proposed the “crescendo effect” model, describing how repeated exposure to morally distressing situations leads to accumulation of “moral residue” with potentially severe consequences for professional wellbeing and patient care [11]. This concept appears relevant in transplantation, where team members repeatedly navigate the boundary between life and death.

Despite Serbia’s opt-out (presumed consent) legislative framework since 2018 [12], clinical practice reveals a striking discrepancy: teams almost invariably seek and require explicit family consent. This gap between legal framework and clinical practice is not unique to Serbia; in several presumed-consent systems, family involvement remains common in practice [13], likely reflecting both cultural expectations and clinicians’ preference for relational rather than purely legal consent.

Moral distress in procurement may therefore stem from two intertwined dimensions: a relational dimension, centred on communication with and the grief of donor families, and a procedural-existential dimension, centred on performing retrieval after death by neurological criteria. The present study measures overall moral distress rather than attempting to isolate these sources. This study addresses these gaps by measuring moral distress among transplant team members at a Serbian tertiary centre, with particular focus on examining differences across professional groups and identifying factors associated with distress intensity. We hypothesised that: (H1) transplant team members experience significant levels of moral distress related to deceased donor organ procurement; (H2) surgeons involved in explantation demonstrate highest distress levels due to their direct procedural role; and (H3) years of experience correlate negatively with moral distress intensity.

## Methods

### Study design and setting

This cross-sectional study was conducted at the University Clinical Centre of Vojvodina, Novi Sad, Serbia’s second-largest transplant centre. The centre’s multidisciplinary transplant team includes vascular surgeons, nephrologists, anaesthesiologists, nurses, and transplant coordinators. Between January 2014 and December 2023, the centre performed 40 deceased donor kidney transplantations (mean 4 per year). The programme is limited to kidney transplantation; multi-organ procurements (liver, heart) occur only when teams from collaborating national centres participate. This rate reflects Serbia’s position among low-donation European countries.

The typical procurement procedure involves a multidisciplinary team responding to donor notification, usually within 4–6 h. The vascular surgery team leads organ retrieval, with anaesthesiology maintaining donor stability, nursing providing intraoperative support, and coordinators managing family communication and logistics. Teams operate on an on-call basis, with procurements occurring at irregular intervals averaging 4–5 per year.

### Participants and sampling

All 28 active transplant team members were invited to participate. Inclusion criteria were: (1) current involvement in deceased donor organ procurement procedures, and (2) minimum one year of transplantation experience. No exclusion criteria were applied beyond failure to meet inclusion criteria. Of 28 eligible professionals, 22 completed the questionnaire (78.6% response rate). The sample included 4 vascular surgeons, 3 anaesthesiologists, 7 nurses, 3 nephrologists, and 5 transplant coordinators. Mean team membership duration was 9.2 ± 5.8 years (range 2–18).

### Instrument

We used the Moral Distress Scale-Revised (MDS-R), originally developed and validated by Hamric et al. [14], adapted for transplantation contexts. The adaptation process involved: (1) forward translation into Serbian by two independent medical translators; (2) back-translation into English by a native English-speaking physician; (3) expert panel review comprising 3 transplant professionals and 2 ethicists; (4) modification of 5 items to reflect transplantation-specific scenarios (e.g., replacing “end-of-life care” with “brain death determination,” “futile care” with “organ viability concerns”); (5) pilot testing with 5 transplant professionals not included in the main study; and (6) final refinement based on pilot feedback. Minor wording changes were made to improve clarity and contextual relevance; no items were added or removed.

The final Serbian-language instrument contained 21 items measuring frequency (0 = never to 4 = very frequently) and intensity (0 = none to 4 = great extent) of moral distress. The MDS-R total score was calculated as the sum of item composite scores (frequency × intensity) across all 21 items, yielding a theoretical range of 0–336. Internal consistency was excellent (Cronbach’s α = 0.89), comparable to the original instrument validation [14]. While internal consistency was satisfactory, the adapted instrument has not undergone full psychometric validation for transplantation contexts. Given the sample size (*n* = 22), confirmatory factor analysis was not performed; the dimensional structure was assumed based on the original validated MDS-R rather than empirically confirmed in this sample. Test-retest reliability was not assessed due to the single-administration design. These limitations affect the interpretability of item-level analyses (Table 3) and should be considered when interpreting results.

### Data collection

Questionnaires were distributed electronically via secure platform between September and November 2023. Participants provided demographic information including age, sex, profession, years of general medical experience, and years specifically in transplantation. Additional items assessed intention to leave transplantation work (three ordinal categories coded 0/1/2: never considered, considered leaving, took concrete steps). A single item on perception of institutional support was included but is not reported here, as the categorical responses did not yield sufficient variability for meaningful analysis in this sample. The questionnaire also included one optional open-ended item inviting participants to describe the most challenging ethical aspect of their procurement experience; responses to this item were not formally analysed but are cited in the Discussion only to contextualise quantitative findings. There were no missing data; all 22 respondents completed every item.

### Statistical analysis

Data were analysed using SPSS version 26.0. Normality was assessed using Shapiro–Wilk test. Although the overall MDS-R total score and years of experience did not deviate significantly from normality, subgroup sizes were very small (*n* = 3–7), making normality tests underpowered and parametric assumptions unreliable; therefore, we used non-parametric tests consistently. Descriptive statistics included means, standard deviations, medians, and interquartile ranges.

Group comparisons across five professional categories used Kruskal–Wallis H test with Dunn’s post-hoc comparisons and Bonferroni correction for 10 pairwise tests. Effect size was calculated using Kruskal–Wallis eta-squared (η² = (H − k + 1)/(n − k)), as commonly reported for non-parametric group comparisons. Correlations were assessed using Spearman’s rho (ρ), accompanied by 95% bootstrap confidence intervals (1,000 resamples); bootstrap intervals were preferred because distributional assumptions for asymptotic interval estimation are less reliable in small samples. Statistical significance was set at *p* < 0.05.

Given the small sample, particularly within some professional groups (*n* = 3–7), the study is underpowered for anything other than large between-group effects. All findings should be interpreted as preliminary, and significant results require confirmation in larger multi-centre studies.

### Ethical considerations

The study was approved by the University Clinical Centre of Vojvodina Ethics Committee (No. 00–15/2023, date: January 12, 2023). All participants provided written informed consent. Anonymity was ensured through assignment of participant codes. Given the small team size, potential identifiability of respondents through professional-group-level reporting was disclosed in the informed consent process. To reduce response bias, the survey was anonymous and completed electronically without supervisors’ access to individual responses.

## Results

### Participant characteristics

Twenty-two of 28 invited transplant team members completed the survey (response rate 78.6%). Mean age was 41.3 ± 8.7 years, with 72.7% female participants. Mean years of transplantation experience was 9.2 ± 5.8 years (range 2–18 years). Detailed demographic characteristics are presented in Table 1. Notably, professional groups differed in demographic composition: vascular surgeons were older (mean 48.2 years), exclusively male, and had more transplantation experience (14.5 years) and higher procedural volume (median 55 procurements) compared with other groups. These between-group demographic differences are relevant when interpreting group-level MDS-R comparisons (see Discussion). Procurement participation counts are reported descriptively and were not used as an independent predictor due to small sample size.

**Table 1:** Demographic and Professional Characteristics of Participants (*n* = 22)

Characteristic	Overall (*n* = 22)	VS (*n* = 4)	AN (*n* = 3)	NR (*n* = 7)	NP (*n* = 3)	TC (*n* = 5)
Age, mean ± SD	41.3 ± 8.7	48.2 ± 6.1	43.7 ± 5.8	38.4 ± 9.2	44.3 ± 7.5	39.6 ± 7.3
Female, n (%)	16 (72.7)	0 (0)	1 (33.3)	7 (100)	2 (66.7)	5 (100)
Yrs med. exp.	16.8 ± 9.2	22.5 ± 5.3	18.3 ± 4.7	13.1 ± 8.9	19.7 ± 6.4	14.2 ± 7.8
Yrs tx exp.	9.2 ± 5.8	14.5 ± 3.8	10.7 ± 4.2	6.3 ± 4.1	11.3 ± 5.6	7.4 ± 5.2
Procurements	25(8–65)	55(40–65)	35(22–48)	18(8–32)	30(15–45)	20(10–40)

Procurements reported as median (range)

*VS *Vascular Surgeons*, AN *Anaesthesiologists*, NR *Nurses*, NP *Nephrologists*, TC *Transplant Coordinators

### Overall moral distress levels

**Fig. 1:**
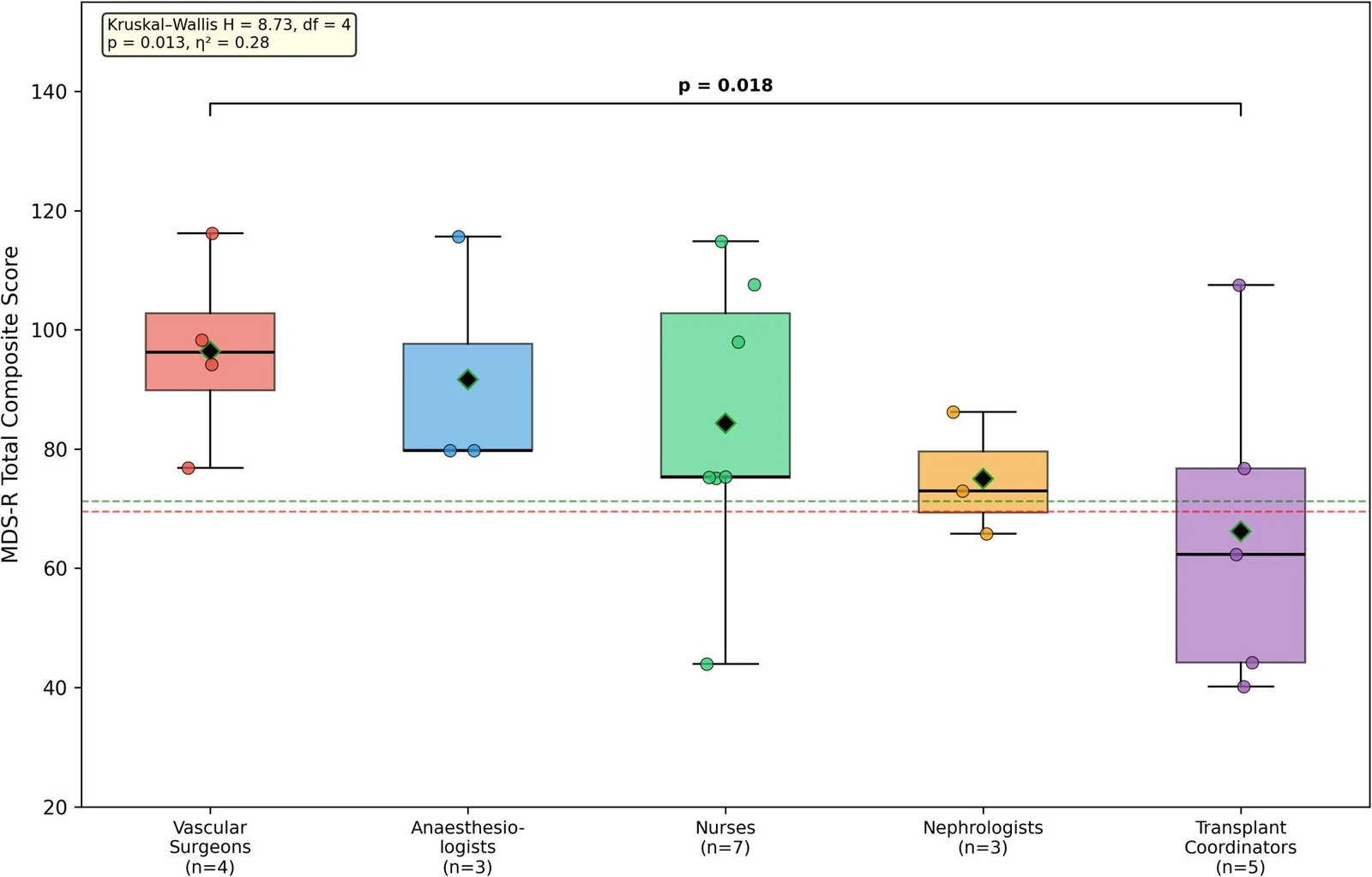
Distribution of Moral Distress Scale-Revised Scores by Professional Group. Box plots show median, interquartile range, and individual data points (n = 22). Diamond indicates group mean. Bracket indicates the only significant post-hoc comparison: vascular surgeons vs. transplant coordinators (adjusted p = 0.018). Dashed horizontal lines show published reference means from other settings for descriptive context only (red: ICU, 69.5 [3]; green: emergency, 71.2 [15]). Kruskal–Wallis H = 8.73, df = 4, p = 0.013, η² = 0.28

The mean MDS-R total composite score was 82.4 ± 31.8 (median 78.5, IQR 58–102), with individual scores ranging from 38 to 132 on the 0–336 theoretical scale. The 95% confidence interval for the sample mean was 68.3–96.5 (SE = 6.8). This indicates substantial moral distress in this sample. Compared with published point estimates from ICU settings (69.5) and emergency settings (71.2), our estimate was numerically higher; however, the 95% CI for our sample mean encompasses both reference values. Accordingly, these cross-study comparisons are descriptive and hypothesis-generating rather than inferential. Fig. 1 illustrates the distribution of scores across professional groups.

### Differences across professional groups

Significant differences in MDS-R scores emerged across professional groups (Kruskal–Wallis H = 8.73, df = 4, *p* = 0.013, η² = 0.28). Vascular surgeons reported the highest observed mean scores (96.4 ± 27.3), followed by anaesthesiologists (91.7 ± 19.8), nurses (84.3 ± 32.1), nephrologists (75.0 ± 29.4), and transplant coordinators (66.2 ± 26.7). Post-hoc analysis using Dunn’s test with Bonferroni correction for 10 pairwise comparisons revealed a significant difference only between vascular surgeons and transplant coordinators (adjusted *p* = 0.018); no other pairwise comparisons reached statistical significance. Accordingly, the overall group effect appears to be driven primarily by the contrast between these two groups, rather than by a consistent gradient across all five professions. Complete results are presented in Table 2.

**Table 2:** Moral distress scale-revised scores by professional group (*n* = 22)

Professional Group	*n*	Mean ± SD	Median (IQR)	Range	Post-hoc
Vascular Surgeons	4	96.4 ± 27.3	98.0 (72–121)	65–132	VS > TC, *p* = 0.018
Anaesthesiologists	3	91.7 ± 19.8	88.0 (74–110)	74–113	—
Nurses	7	84.3 ± 32.1	82.0 (58–112)	38–128	—
Nephrologists	3	75.0 ± 29.4	72.0 (48–102)	48–105	—
Transplant Coord.	5	66.2 ± 26.7	62.0 (44–88)	42–98	—

Kruskal–Wallis H = 8.73, df = 4, *p* = 0.013, η² = 0.28. Post-hoc: Dunn’s test with Bonferroni correction (10 pairwise comparisons). Overall 95% CI for sample mean: 68.3–96.5. Per-group CIs not reported as subgroup sizes (*n* = 3–7) are too small for stable estimation

### Top-ranked MDS-R items

Analysis of individual MDS-R items revealed highest composite scores (frequency × intensity) for items related to “time pressure in brain death diagnosis” (8.5 ± 2.6), “family communication dilemmas” (8.4 ± 3.0), and “donor body objectification concerns” (7.6 ± 3.3). The highest intensity scores were for items addressing “family communication dilemmas” (3.5 ± 0.8) and “donor body objectification” (3.4 ± 0.9), while frequency scores were highest for “time pressure in brain death diagnosis” (2.8 ± 0.7) and “institutional pressures for efficiency” (2.4 ± 0.6). Notably, the two highest-intensity items reflect concerns shared across all professional groups, not solely those performing surgical retrieval. Table 3 summarises the top-ranked items by composite score; the full 21-item table is available as Additional file 2. Thematic group labels are descriptive and do not imply confirmed factorial dimensions, as factor analysis was not feasible with the current sample size.

**Table 3:** Top-ranked MDS-R items by composite score (*n* = 22)

Item	Thematic group	Freq.	Intensity	Composite	Rank
Time pressure in brain death diagnosis	Diagnostic	2.8 ± 0.7	3.2 ± 0.7	8.5 ± 2.6	1
Family communication dilemmas	Communication	2.4 ± 0.9	3.5 ± 0.8	8.4 ± 3.0	2
Donor body objectification concerns	Ethical	2.2 ± 0.8	3.4 ± 0.9	7.6 ± 3.3	3
Institutional pressures for efficiency	Institutional	2.4 ± 0.6	2.9 ± 0.8	7.1 ± 2.7	4
Conflict with family wishes	Communication	1.8 ± 0.8	2.9 ± 0.9	5.5 ± 2.9	5

Shown are the 5 highest-ranked items; full 21-item table available in Additional file 2. Thematic group labels assigned by authors based on item content; groupings are descriptive and do not represent confirmed factorial dimensions. Frequency scale 0–4; Intensity scale 0–4; Composite = Frequency × Intensity (range 0–16 per item)

### Correlation with experience

**Fig. 2:**
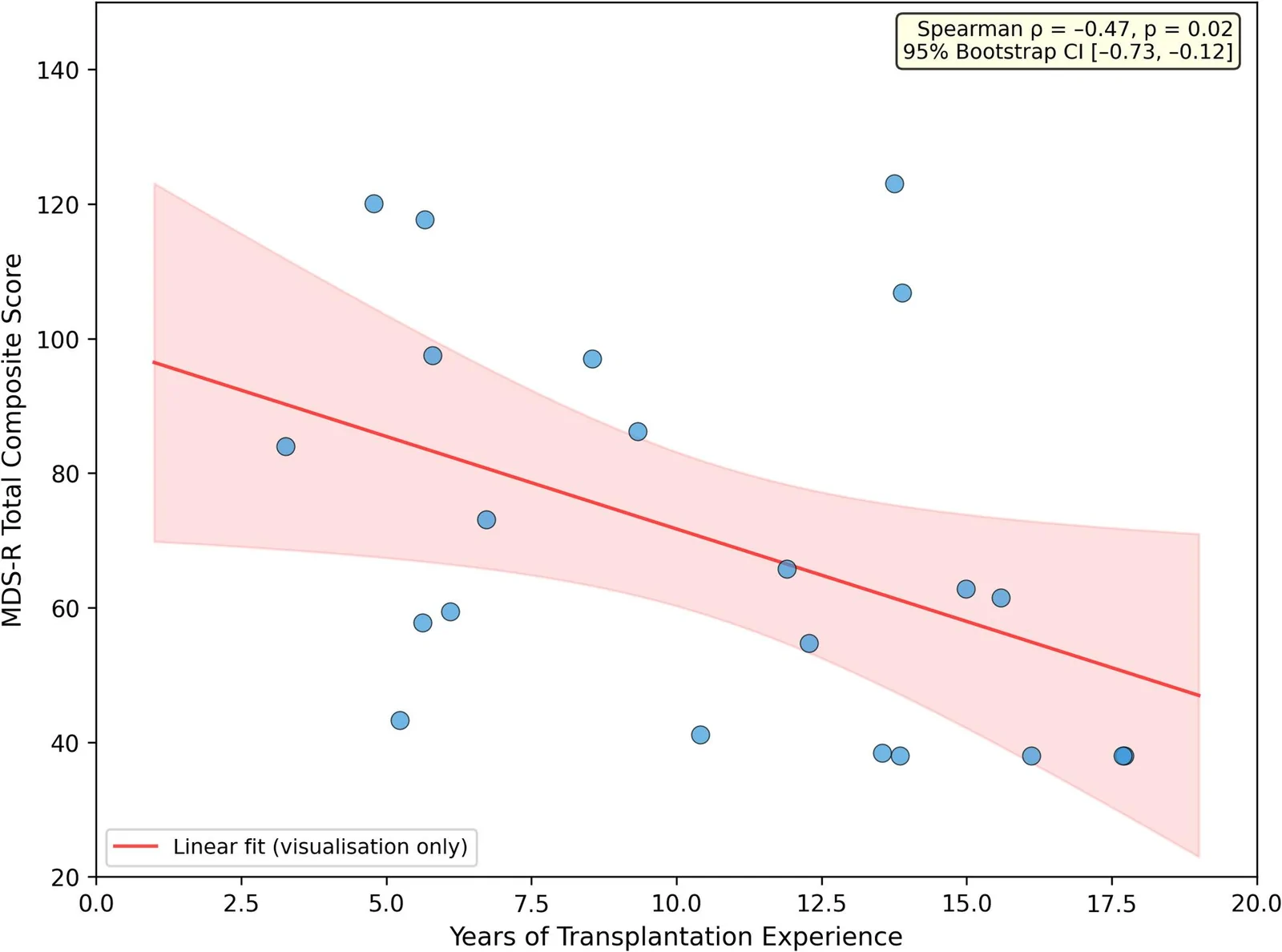
Correlation Between Years of Transplantation Experience and Moral Distress Scores. Scatter plot with linear regression line (for visualisation only) and 95% confidence band. Each point represents one participant (n = 22). Primary inference: Spearman’s ρ = –0.47, 95% bootstrap CI [–0.73, –0.12], p = 0.02

Years of transplantation experience showed significant negative correlation with MDS-R scores (Spearman’s ρ = − 0.47, 95% bootstrap CI [–0.73, − 0.12], *p* = 0.02). For visualisation purposes, a linear regression line is included in Fig. 2 for visualisation only; the primary inference is based on the non-parametric Spearman correlation.

### Intention to leave and career impact

**Fig. 3:**
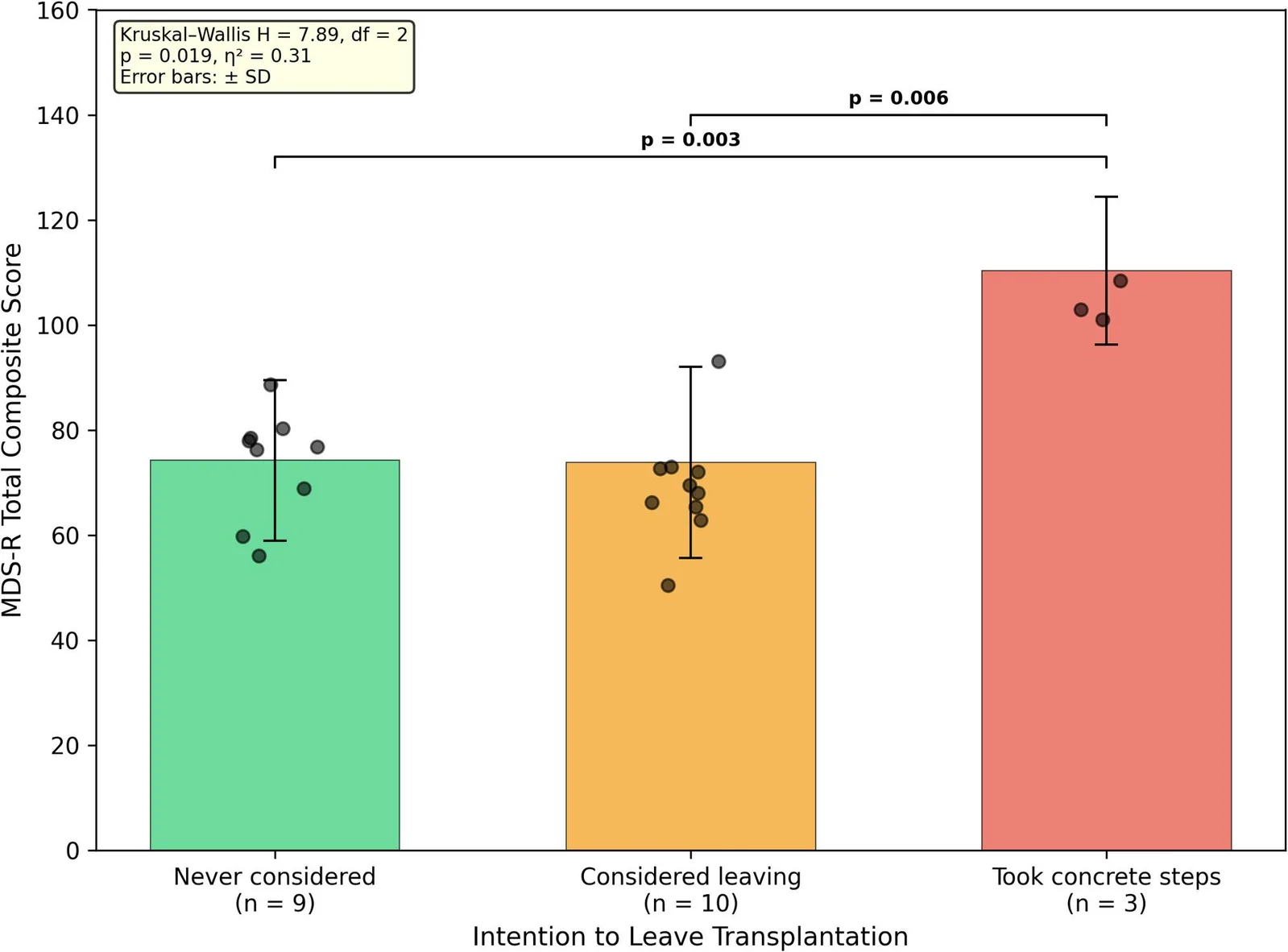
Relationship Between Moral Distress Scores and Intention to Leave Transplantation. Mean MDS-R scores (± SD) by intention category with individual data points. Kruskal–Wallis H = 7.89, df = 2, p = 0.019, η² = 0.31. Post-hoc Dunn’s test with Bonferroni correction: “Took concrete steps” vs. “Never considered” p = 0.003; vs. “Considered leaving” p = 0.006

Regarding career intentions, 40.9% of participants (*n* = 9) had never considered leaving transplantation, 45.5% (*n* = 10) had considered leaving, and 13.6% (*n* = 3) had taken concrete steps toward career change. MDS-R scores differed significantly across these groups (Kruskal–Wallis H = 7.89, df = 2, *p* = 0.019, η² = 0.31): never considered (mean 74.2 ± 15.3), considered leaving (mean 73.8 ± 18.2), and took concrete steps (mean 110.3 ± 14.1). Notably, the significant effect was driven primarily by the “took concrete steps” group (*n* = 3); the “never considered” and “considered leaving” groups had nearly identical mean scores. Intention to leave, coded as an ordinal variable (0/1/2), correlated with moral distress scores (Spearman’s ρ = 0.52, *p* = 0.01; bootstrap 95% CI [0.18, 0.76]). The questionnaire did not systematically collect reasons for considering departure; therefore, this association should be interpreted as an exploratory signal rather than evidence of a specific causal pathway. Post-hoc comparisons involving the “took concrete steps” group (*n* = 3) should be interpreted with particular caution given the very small subgroup size. Post-hoc Dunn’s test with Bonferroni correction: “took concrete steps” vs. “never considered” *p* = 0.003; vs. “considered leaving” *p* = 0.006. (Fig. 3)

### Comparison with other clinical settings

To provide descriptive context, Table 4 presents MDS-R scores from published studies of other high-stakes clinical settings alongside our findings. These comparisons are strictly descriptive given differences in populations, instruments, countries, and cultural contexts, and do not permit formal statistical inference.

**Table 4:** Descriptive comparison of MDS-R scores across clinical settings

Setting	Population	*n*	MDS-*R* Mean ± SD	Country	Ref
This study	Procurement team	22	82.4 ± 31.8	Serbia	—
ICU nurses	ICU subgroup	592	69.5 ± 30.5	USA	[3]
Healthcare prof.	Institution-wide	592	76.0 ± 42.4	USA	[3]
Emergency nurses	ED nurses	51	71.2 ± 28.4	USA	[15]
ICU nurses	ICU nurses	153	72.4 ± 53.6	Iran	[16]

Descriptive context only; direct statistical comparison not possible. All values are total composite MDS-R scores (sum of frequency × intensity). Both rows from [3] refer to subgroups within the same institution-wide survey. Oldenmenger et al. [17] reported mean 36.4 ± 26.0 among 654 Dutch general-ward nurses

## Discussion

### Principal findings

To our knowledge, this is the first dedicated cross-sectional assessment of moral distress among transplant procurement team members in Serbia. The mean MDS-R total composite score of 82.4 (95% CI 68.3–96.5) suggests substantial moral distress in this sample, consistent with our first hypothesis. While the point estimate was numerically higher than published means from ICU (69.5 [3]) and emergency settings (71.2 [15]), the confidence interval encompasses both reference values, precluding claims about between-setting differences. These comparisons are descriptive only.

Within this small single-centre sample, vascular surgeons had the highest observed moral distress scores (96.4 ± 27.3). Several interpretations should be considered. First, the finding may reflect role-specific exposure: surgeons directly perform organ retrieval from donors with maintained physiological support, which may impose a distinct psychological burden. Second, however, vascular surgeons in our sample were also older, more experienced, exclusively male, and had higher procedural volumes (Table 1). Without multivariable analysis—which was not feasible given *n* = 4 in this subgroup—we cannot determine whether profession per se, or these correlated demographic and experiential factors, account for the observed difference. Third, selection effects may play a role: professionals with lower distress tolerance may leave before accumulating extensive experience, producing a survivorship effect that confounds the group comparison. Fourth, the significant post-hoc contrast was limited to vascular surgeons versus transplant coordinators; the three intermediate groups did not differ significantly, suggesting a contrast between the two most distinct roles rather than a consistent gradient across all professions. Accordingly, we interpret this finding as hypothesis-generating and requiring confirmation in larger, multi-centre samples with multivariable adjustment.

An important observation is that the highest-intensity distress items across the full sample—family communication dilemmas and donor body objectification—reflect concerns shared by all professional groups, not solely surgeons. Although surgeons typically do not lead family communication, they are exposed to the emotional context of the procurement environment, including awareness of family distress and the broader ethical tensions surrounding the procedure. Additionally, the “donor body objectification” item likely resonates particularly with team members who physically handle the donor. Future studies with larger samples should examine item-level scores by professional group to clarify whether distress profiles differ qualitatively across roles or primarily differ in overall magnitude. Consistent with this, our companion qualitative study at the same centre [18] identified distress arising both from the relational burden of navigating family grief and from the procedure itself, including persistent unease about brain death as death, supporting the view that these sources coexist rather than being mutually exclusive.

The significant negative correlation between experience and distress (Spearman’s ρ = − 0.47, *p* = 0.02) is consistent with our third hypothesis and with Rushton’s concept of “moral resilience” [19]. Rushton defines moral resilience as the capacity of an individual to sustain or restore their integrity in response to moral adversity, including the ability to respond to moral distress in ways that preserve professional values and wellbeing [19]. In our data, the inverse association between experience and distress is compatible with the development of such resilience over time. However, as noted above for the professional-group comparison, the cross-sectional design cannot distinguish genuine resilience from selective attrition, whereby the most distressed clinicians may leave the field before accumulating experience.

### Comparison with existing literature

Our findings are consistent with the “crescendo effect” model proposed by Epstein and Hamric [11], wherein repeated exposure to morally distressing situations without adequate support leads to accumulated moral residue. The correlation between distress and intention to leave (Spearman’s ρ = 0.52) parallels findings from institution-wide surveys showing moral distress as a significant predictor of turnover intention [16]. Directly comparable data from procurement teams are scarce, and the available studies are largely qualitative or use heterogeneous instruments: a study of nurses in solid-organ transplant units linked moral distress to disregard for patient autonomy and therapeutic obstinacy [20], and qualitative work with transplant coordinators described an intense, sustained emotional burden tied to family interaction and consent [21]. We are not aware of prior quantitative MDS-R data from organ procurement teams in Serbia; the comparators in Table 4 therefore derive from intensive care and emergency settings and provide descriptive context only.

The gap between Serbia’s opt-out legislation and clinical practice—with teams commonly seeking explicit family consent—is consistent with patterns reported in other presumed-consent systems [13]. This practice adds a communication burden that may contribute to the high intensity scores observed for family communication dilemmas.

### Intention to leave

The association between moral distress and self-reported intention to leave transplantation should be interpreted cautiously. Although the correlation was statistically significant (Spearman’s ρ = 0.52), the statistical effect was driven primarily by three participants who reported taking concrete steps toward career change. The two larger groups (“never considered” and “considered leaving”) had nearly identical mean MDS-R scores. Moreover, intention to leave was measured by a single ordinal item without established psychometric properties, and specific reasons for considering departure were not systematically collected. This limits our ability to establish whether moral distress was the primary driver of turnover intention, or whether other factors (e.g., workload, compensation, career opportunities) contributed. In the optional open-ended item, some participants noted tensions between efficiency demands and thoroughness; these comments were not formally analysed and are mentioned only as contextual observations. Future studies should examine turnover intention with validated instruments and a larger sample.

### Clinical implications

Although this was an exploratory single-centre study, several areas appear suitable for further investigation. Potential strategies for evaluation include structured post-procurement debriefings, accessible ethics consultation, and peer support programmes. Additionally, the underlying causes of moral distress—including time pressure, family communication dilemmas, and institutional factors—suggest that organisational-level interventions addressing team management and communication protocols may deserve examination. These options should be considered as quality-improvement hypotheses rather than definitive practice guidance, given the exploratory single-centre design.

### Strengths and limitations

Strengths of this study include use of an established instrument with demonstrated internal reliability in our Serbian-language adaptation, high response rate (78.6%), and focus on an understudied population in a low-donation country. The inclusion of all professional groups involved in procurement provides comprehensive team-level data.

Several important limitations must be acknowledged. First, the cross-sectional design prevents causal inference. Second, this is a single-centre study in a specific cultural and legislative context (Serbia; low donation rate; opt-out legislation not routinely applied in practice), limiting generalisability. Third, the sample size (*n* = 22) constrains statistical power, particularly for subgroup analyses with groups as small as *n* = 3–4; all findings should therefore be interpreted as exploratory. Fourth, professional groups differed substantially in demographic composition (age, sex, experience, procedural volume); without multivariable adjustment, observed group differences in MDS-R scores may be confounded by these factors. Fifth, although internal consistency was excellent (α = 0.89), other measurement properties of the adapted instrument (e.g., test-retest reliability, construct validity, content validity index) could not be evaluated due to sample size; measurement uncertainty may have affected effect estimates. Sixth, intention to leave was assessed by a single ordinal item without established psychometric properties, and reasons for considering departure were not systematically collected. Seventh, given the small team size, potential identifiability of respondents despite anonymisation procedures may have influenced responses toward social desirability. Eighth, comparisons with published MDS-R scores (Table 4) are descriptive only, as differences in instrument versions, scoring methods, populations, and cultural contexts limit comparability. Future multi-centre studies with larger samples should address these limitations.

## Conclusions

Organ procurement team members at this Serbian centre reported substantial moral distress as measured by a transplantation-adapted MDS-R. While vascular surgeons had the highest observed scores, this finding may reflect role-specific factors, demographic differences between professional groups, or other unmeasured confounders, and requires confirmation with larger samples and multivariable analysis. The association between moral distress and self-reported intention to leave transplantation, though exploratory, suggests that structured support strategies for procurement teams in low-donation settings warrant formal evaluation in future multicentre studies; the effectiveness of specific interventions remains to be established. Future studies should use multicentre and, where feasible, longitudinal designs to test whether these associations persist in larger samples and to clarify temporal relationships between distress and intention to leave.

## Supplementary Information

Additional file 1: STROBE checklist for cross-sectional studies. (DOCX).

Additional file 2: Complete 21-item MDS-R analysis with all items ranked by composite score. (DOCX).

## Data Availability

The datasets used and/or analysed during the current study are available from the corresponding author on reasonable request.

## Abbreviations

CI: Confidence interval
EU: European Union
H: Kruskal–Wallis test statistic
ICU: Intensive care unit
IQR: Interquartile range
MDS-R: Moral Distress Scale-Revised
pmp: Per million population
SD: Standard deviation
SE: Standard error
SPSS: Statistical Package for the Social Sciences
STROBE: Strengthening the Reporting of Observational Studies in Epidemiology

## References

[1] Jameton A. Nursing Practice: The Ethical Issues. Englewood Cliffs, NJ: Prentice Hall; 1984.

[2] Corley MC, Elswick RK, Gorman M, Clor T. Development and evaluation of a moral distress scale. J Adv Nurs. 2001;33(2):250–6. 10.1046/j.1365-2648.2001.01658.x. 11168709

[3] Whitehead PB, Herbertson RK, Hamric AB, Epstein EG, Fisher JM. Moral distress among healthcare professionals: report of an institution-wide survey. J Nurs Scholarsh. 2015;47(2):117–25. 10.1111/jnu.12115. 25440758

[4] Austin CL, Saylor R, Finley PJ. Moral distress in physicians and nurses: impact on professional quality of life and turnover. Psychol Trauma. 2017;9(4):399–406. 10.1037/tra0000201. 27797570

[5] European Directorate for the Quality of Medicines & HealthCare (EDQM). Newsletter Transplant: International Figures on Donation and Transplantation 2023. Strasbourg: Council of Europe; 2024.

[6] Global Observatory on Donation and Transplantation. International Report on Organ Donation and Transplantation Activities 2023. Geneva: World Health Organization; 2024.

[7] Greer DM, Shemie SD, Lewis A, et al. Determination of brain death/death by neurological criteria: the World Brain Death Project. JAMA. 2020;324(11):1078–97. 10.1001/jama.2020.11586. 32761206

[8] Shemie SD, Hornby L, Baker A, et al. International guideline development for the determination of death. Intensive Care Med. 2014;40(6):788–97. 10.1007/s00134-014-3242-7. PMC4028548 24664151

[9] Truog RD, Berlinger N, Zacharias RL, et al. Brain death at fifty: exploring consensus, controversy, and contexts. Hastings Cent Rep. 2018;48(Suppl 4):S2–5. 10.1002/hast.942. 30584863

[10] Regehr C, Kjerulf M, Popova SR, Baker AJ. Trauma and tribulation: the experiences and attitudes of operating room nurses working with organ donors. J Clin Nurs. 2004;13(4):430–7. 10.1111/j.1365-2702.2004.00905.x. 15086629

[11] Epstein EG, Hamric AB. Moral distress, moral residue, and the crescendo effect. J Clin Ethics. 2009;20(4):330–42. PMID: 20120853. 20120853

[12] Republic of Serbia. Law on Human Organ Transplantation for the Purpose of Medical Treatment. Official Gaz Repub Serbia. 2018;57:14–29. [in Serbian].

[13] Molina-Pérez A, Rodríguez-Arias D, Delgado-Rodríguez J, et al. Public knowledge and attitudes towards consent policies for organ donation in Europe: a systematic review. Transpl Rev. 2019;33(1):1–8. 10.1016/j.trre.2018.09.001. 30318183

[14] Hamric AB, Borchers CT, Epstein EG. Development and testing of an instrument to measure moral distress in healthcare professionals. AJOB Prim Res. 2012;3(2):1–9. 10.1080/21507716.2011.652337.

[15] Fernandez-Parsons R, Rodriguez L, Goyal D. Moral distress in emergency nurses. J Emerg Nurs. 2013;39(6):547–52. 10.1016/j.jen.2012.12.009. 23414814

[16] Shoorideh FA, Ashktorab T, Yaghmaei F, Alavi Majd H. Relationship between ICU nurses’ moral distress with burnout and anticipated turnover. Nurs Ethics. 2015;22(1):64–76. 10.1177/0969733014534874. 24948793

[17] Oldenmenger WH, Berger E, Stoppelenburg A, et al. The inner struggle of nurses, exploring moral distress among hospital nurses: a cross-sectional study. Int J Nurs Stud. 2024;158:104864. 10.1016/j.ijnurstu.2024.104864. 39094220

[18] Nikolić D, Pasternak J, Manojlović V, Budinski S, Basta Nikolić M, Batinić N. The weight of witness: ethical issues in organ procurement. Nurs Ethics. 2026. 10.1177/09697330261449303. 42209412

[19] Rushton CH. Moral resilience: a capacity for navigating moral distress in critical care. AACN Adv Crit Care. 2016;27(1):111–9. 10.4037/aacnacc2016275. 26909461

[20] Victorino JP, de Brito ES, Westin UM, Vargas MAO, Mendes KDS, Ventura CAA. Health advocacy and moral distress among nurses in organ transplant units. Nurs Ethics. 2024;32(3):994–1003. 10.1177/09697330241295368. 39443290

[21] Tarabeih M, Bokek-Cohen Y. Between health and death: the intense emotional pain experienced by transplant nurses. Nurs Inq. 2020;27(2):e12335. 10.1111/nin.12335. 31960534

