# Supplementary material for: Moral distress and turnover intention in deceased donor organ procurement teams in Serbia: a cross-sectional study
Source: BMC Med Ethics. 2026 Jun 29;27:209. doi: 10.1186/s12910-026-01533-0 (PMC13621484; doi:10.1186/s12910-026-01533-0)
Supplement: Supplementary file 1 [file 12910_2026_1533_MOESM1_ESM.docx]

**STROBE Statement—Checklist of items for cross-sectional studies**

*Additional file 1 for: Nikolić D et al. Moral Distress and Turnover Intention in Deceased Donor Organ Procurement Teams in Serbia: A Cross-Sectional Study*

| **Item** | **STROBE Recommendation** | **Reported on** | **Comment** |
| --- | --- | --- | --- |
| 1 | Title and abstract: (a) study design in title; (b) informative abstract | 1, Abstract | Title includes 'Cross-Sectional Study'; structured abstract with Background/Methods/Results/Conclusions |
| 2 | Background/rationale: scientific background and rationale | Background | Paragraphs 1–5 explain moral distress concept, transplantation context, gaps |
| 3 | Objectives: specific objectives and hypotheses | Background | Final paragraph: H1, H2, H3 stated explicitly |
| 4 | Study design: key elements of study design | Methods | Cross-sectional design stated in first sentence |
| 5 | Setting: setting, locations, relevant dates | Methods 2.1 | University Clinical Centre of Vojvodina; Sept–Nov 2023 |
| 6 | Participants: eligibility criteria, sources, methods of selection | Methods 2.2 | All 28 active members invited; inclusion criteria listed; 22 completed |
| 7 | Variables: outcomes, predictors, confounders | Methods 2.3–2.4 | MDS-R composite score; profession, experience, intention to leave |
| 8 | Data sources/measurement: describe instrument | Methods 2.3 | MDS-R adaptation process (6 steps), 21 items, scoring described |
| 9 | Bias: describe efforts to address bias | Methods 2.4, 2.6 | Anonymous electronic survey; no supervisor access; response bias noted |
| 10 | Study size: explain how study size arrived at | Methods 2.2, 2.5 | Census of all 28 eligible; n=22; power limitations acknowledged |
| 11 | Quantitative variables: how handled in analyses | Methods 2.5 | Non-parametric throughout; MDS-R composite; ordinal intention variable |
| 12 | Statistical methods: (a–e) all statistical methods described | Methods 2.5 | Kruskal–Wallis, Dunn’s post-hoc, Bonferroni, Spearman, bootstrap CI |
| 13 | Participants: numbers at each stage; reasons for non-participation | Results 3.1 | 22/28 (78.6%); 6 non-responders not characterised |
| 14 | Descriptive data: characteristics of participants | Table 1 | Age, sex, experience, procurements by professional group |
| 15 | Outcome data: numbers of outcome events or summary measures | Results 3.2 | Mean 82.4 ± 31.8; median, IQR, range, 95% CI reported |
| 16 | Main results: (a) unadjusted estimates with CI; (b) adjusted; (c) translations | Results 3.3–3.6 | Kruskal–Wallis with effect size; Spearman with bootstrap CI; post-hoc p-values |
| 17 | Other analyses: e.g. subgroup, interaction, sensitivity | Results 3.4, 3.7 | Item-level analysis (Table 3); cross-study comparison (Table 4) |
| 18 | Key results: summarise with reference to objectives | Discussion 4.1 | All three hypotheses addressed with evidence and caveats |
| 19 | Limitations: discuss limitations and potential bias | Discussion 4.4 | Eight limitations discussed: cross-sectional design, single-centre setting, small sample size, demographic confounding between groups, incomplete psychometric validation, single-item turnover measure, possible identifiability/social desirability bias, limited cross-study comparability |
| 20 | Interpretation: cautious interpretation considering objectives, limitations, multiplicity | Discussion 4.1–4.2 | Exploratory framing throughout; descriptive comparisons only |
| 21 | Generalisability: discuss generalisability | Discussion 4.4 | Specific cultural/legislative context acknowledged; multicentre studies recommended |
| 22 | Funding: give source of funding and role of funders | Declarations | No funding received |
