# Supplementary material for: Moral distress and turnover intention in deceased donor organ procurement teams in Serbia: a cross-sectional study
Source: BMC Med Ethics. 2026 Jun 29;27:209. doi: 10.1186/s12910-026-01533-0 (PMC13621484; doi:10.1186/s12910-026-01533-0)
Supplement: Supplementary file 2 [file 12910_2026_1533_MOESM2_ESM.docx]

**Additional file 2**

**Complete Moral Distress Scale-Revised Item Analysis: All 21 Items Ranked by Composite Score (n = 22)**

| **MDS-R Item** | **Thematic Group** | **Freq.** | **Intensity** | **Composite** | **Rank** |
| --- | --- | --- | --- | --- | --- |
| Time pressure in brain death diagnosis | Diagnostic | 2.8 ± 0.7 | 3.2 ± 0.7 | 8.5 ± 2.6 | 1 |
| Family communication dilemmas | Communication | 2.4 ± 0.9 | 3.5 ± 0.8 | 8.4 ± 3.0 | 2 |
| Donor body objectification concerns | Ethical | 2.2 ± 0.8 | 3.4 ± 0.9 | 7.6 ± 3.3 | 3 |
| Institutional pressures for efficiency | Institutional | 2.4 ± 0.6 | 2.9 ± 0.8 | 7.1 ± 2.7 | 4 |
| Conflict with family wishes | Communication | 1.8 ± 0.8 | 2.9 ± 0.9 | 5.5 ± 2.9 | 5 |
| Inadequate support from colleagues | Team | 1.7 ± 0.9 | 2.7 ± 1.0 | 4.7 ± 3.2 | 6 |
| Legal/documentation concerns | Institutional | 2.0 ± 0.7 | 2.3 ± 0.8 | 4.7 ± 2.4 | 7 |
| Resource limitations | Institutional | 1.9 ± 0.8 | 2.2 ± 0.9 | 4.3 ± 2.6 | 8 |
| Disagreement with team decisions | Team | 1.4 ± 0.7 | 2.5 ± 0.8 | 3.6 ± 2.2 | 9 |
| Cultural/religious conflicts | Ethical | 1.3 ± 0.9 | 2.5 ± 1.1 | 3.4 ± 2.8 | 10 |
| Uncertainty about brain death criteria | Diagnostic | 1.2 ± 0.8 | 2.4 ± 1.0 | 2.9 ± 2.5 | 11 |
| Performing procedures against beliefs | Ethical | 1.1 ± 0.9 | 2.4 ± 1.1 | 2.8 ± 2.7 | 12 |
| Inadequate training for ethical aspects | Institutional | 1.3 ± 0.7 | 2.0 ± 0.9 | 2.7 ± 2.1 | 13 |
| Witnessing family distress | Communication | 1.5 ± 0.8 | 1.7 ± 0.7 | 2.7 ± 1.9 | 14 |
| Pressure to expedite procurement | Institutional | 1.2 ± 0.7 | 2.1 ± 0.8 | 2.7 ± 2.0 | 15 |
| Compromised informed consent process | Ethical | 1.0 ± 0.8 | 2.2 ± 1.0 | 2.4 ± 2.4 | 16 |
| Role ambiguity during procurement | Team | 1.1 ± 0.7 | 1.9 ± 0.8 | 2.3 ± 1.8 | 17 |
| Lack of debriefing after difficult cases | Team | 1.2 ± 0.9 | 1.7 ± 0.9 | 2.2 ± 2.2 | 18 |
| Media/public scrutiny concerns | Institutional | 0.8 ± 0.7 | 1.8 ± 1.0 | 1.6 ± 1.8 | 19 |
| Hierarchical pressure from senior staff | Team | 0.7 ± 0.6 | 1.6 ± 0.9 | 1.3 ± 1.4 | 20 |
| Routine desensitisation to donor dignity | Ethical | 0.6 ± 0.6 | 1.4 ± 0.8 | 1.0 ± 1.1 | 21 |

*Note: Frequency scale 0–4 (never to very frequently); Intensity scale 0–4 (none to great extent). Composite = Frequency × Intensity (range 0–16 per item). The sum of item-level mean composites equals the reported overall MDS-R mean (82.4). Items ranked by composite score. Thematic group labels are descriptive, assigned by the authors; they do not represent empirically confirmed factorial dimensions. Top 5 items are presented in Table 3 of the main manuscript.*
